# The Clinical Clues of Pulmonary Alveolar Proteinosis: A Report of 11 Cases and Literature Review

**DOI:** 10.1155/2016/4021928

**Published:** 2016-04-26

**Authors:** Qiongya Mo, Bingbin Wang, Nian Dong, Lianmin Bao, Xiaoqiong Su, Yuping Li, Chengshui Chen

**Affiliations:** Department of Respiratory and Critical Care Medicine, The First Affiliated Hospital of Wenzhou Medical University, Wenzhou, Zhejiang 325000, China

## Abstract

Pulmonary alveolar proteinosis (PAP) is a rare interstitial lung disease characterized by the abnormal alveolar accumulation of surfactant components. The diagnosis of PAP can be easily missed since it is rare and lacks specific clinical symptoms. It is of great importance to have a better understanding of the crucial clue to clinically diagnose PAP and take PAP into consideration in the differential diagnosis of interstitial pulmonary diseases or other diseases with similar manifestations. Here, we analyze the clinical characteristics of 11 cases of PAP patients in local hospital and review the relevant literature in order to provide more information in diagnosis and management of PAP. In our observation, cyfra21-1 and neuron-specific enolase (NSE) known as tumor markers probably can be useful serum markers for diagnosis of PAP. As for the method of pathologic diagnosis, open-lung biopsy was the gold standard but now it is less required because findings on examination of bronchoalveolar lavage fluid (BALF) can help to make the diagnosis. We also have deep experience about when and how to carry out lung lavage.

## 1. Introduction

Pulmonary alveolar proteinosis (PAP), which was first described in 1958 by Rosen et al. [1], is an extremely rare disorder. The annual incidence and prevalence of PAP are 0.36–0.49 and 3.7–6.2 cases per million population, respectively [2, 3]. There are three main forms of PAP: autoimmune, secondary, and congenital PAP, and autoimmune (also called acquired or idiopathic) PAP accounts for almost 90% of PAP cases [4].

PAP is characterized by accumulation of surfactant lipids and proteins, amorphous, eosinophilic, and periodic acid Schiff (PAS) positive materials, in endoalveolar space [1]. Although symptoms of PAP are nonspecific, radiograph signs especially high resolution computed tomography (HRCT) scans are often greatly suggestive of PAP [5]. Lung biopsy and BALF examinations can make the final diagnosis. Moreover, the test of autoantibodies anti-GM-CSF is necessary for the diagnosis of autoimmune PAP. Congenital PAP is caused by mutations in genes coding for surfactant protein and the GM-CSF receptor. Diagnosis of secondary PAP is established on the history of hematologic or solid malignancies, inhalation of inorganic agents, chemotherapy treatment, opportunistic infections, and lysinuric protein intolerance. Lamellar bodies can be shown on electron microscopic examination [6]. Although in the latest two decades researches reported that therapy with granulocyte macrophage-colony stimulating factor (GM-CSF) may be efficient, it is still not commonly used clinically. Whole lung lavage is still the standard and most effective proven therapy.

The clinic course of PAP is variable, ranging from spontaneous resolution to respiratory failure, even death. In the present study, we retrospectively reviewed 11 cases of PAP patients from 2005 to 2014 in our hospital to provide more information in clinical management of PAP patients.

## 2. Clinical Data

11 cases of PAP patients (7 male, 4 female) were retrospectively analyzed, detailed in Tables 1 and 2. The youngest was 30 years old and the oldest was 66 years old. The median age at the time of diagnosis was 50 years. Seven cases had a history of smoking and 2 cases had a history of dust exposure, all of whom were male.

The most common clinical symptom was progressive dyspnea of gradual onset (10/11). Other features included cough (8/11), fever (4/11), and chest pain (2/11). Besides, there was one case who had no discomfort. The findings on physical examination were unremarkable, including inspiratory crackles (6/11), digital clubbing (5/11), and cyanosis (2/11) and 2 cases' physical examination was normal.

As for the laboratory investigations, routine blood counts and the results of routine blood chemical analysis were normal when admitted to hospital. C-reactive protein (CRP) and erythrocyte sedimentation rate (ESR) slightly were elevated in 3 and 5 cases, respectively. The arterial oxygen pressure of 8 cases was lower than 70 mmHg. Seven cases showed an elevation of the serum level of lactate dehydrogenase (LDH). Eight cases showed increase in carcinoembryonic antigen (CEA) level. For the levels of cyfra21-1 and neuron-specific enolase (NSE), 7 cases showed an increase each and 3 cases had no recorded data.

On pulmonary function, 6 cases showed a restrictive ventilator defect. One case showed a combination of restrictive and obstructive ventilator dysfunction. And 4 cases' ventilation function was normal. 11 cases all showed a reduction of the carbon monoxide diffusing capacity (DLCO).

Radiological examinations included chest radiograph or CT scan. 10 cases revealed widespread bilateral airspace disease. There was one case showing asymmetrical single right lung consolidation. Eight cases showed the typical appearance of crazy paving pattern. Three cases presented patchy, ground-glass opacifications.

Diagnosis was established by lung biopsy and/or bronchoalveolar lavage fluid. Five cases received the surgical lung biopsy and among them 4 cases were confirmed. Four cases received transbronchial lung biopsy and only one case was confirmed. The BAL fluid of all cases had an opaque, milky, or yellow muddy appearance. 11 cases received the examination of BAL fluid and 9 cases found the PAS positive material.

For the clinical management, we treated the patients with WLL when one of following conditions appeared: (1) severe dyspnea, cough or chest pain, and significant limitation in daily or sport activities; (2) presence of persistent or progressive respiratory failure; (3) absence of respiratory difficulty at rest, but presence of exercise desaturation (>5% points); (4) repeated pulmonary infection induced by PAP. Six cases were treated by bilateral whole lung lavage. Two cases received incomplete single right lung lavage because one refused the treatment of the other single lung and the other case only had pathological changes in the right lung and both had problem in drainage. Two cases did not receive WLL because of being free of or having slight dyspnea. One case refused WLL treatment for severe pulmonary infection and economic reason. All of the patients who were treated by WLL had clinical reliefs as well as increases of DLCO in different degrees, showing improvements in diffuse capacity. It is worth mentioning that we followed up the two patients who refused WLL for the reason that one was slightly dyspnea and the other had no difficulty in breathing. Then we found that several years after discharge both of the two patients had no symptom and CT scan showed obvious relief. The detail information of DLCO before and after WLL is showed in Table 3.

## 3. Discussion

The diagnosis of pulmonary alveolar proteinosis should be established on the basis of combined examinations of clinical symptoms, lung function, radiology, and evidence of the accumulation of surfactant lipids and proteins in alveolar spaces and macrophages. As PAP is a rare lung disease, it is difficult for clinical physicians to make quick and accurate diagnosis. Therefore it is of great importance to seek for good clinical clues helping improve the accuracy of clinical and pathological diagnosis. In our present study, all the cases were clinically suspected and finally pathologically and/or cytologically diagnosed.

The clinical presentation of PAP is nonspecific. The most common symptom is progressive exertional dyspnea of gradual onset [7]. Cough, fever, chest pain, and hemoptysis can also occur. Physical examination includes inspiratory crackles (50 percent of patients), cyanosis (25 percent), and digital clubbing in a small percentage [4]. But in our study, digital clubbing is a little more common than cyanosis maybe because of relatively long duration of disease and chronic hypoxia.

In laboratory investigation, elevated serum levels of LDH [8], tumor markers [9–11] including CEA, cyfra21-1, and NSE, and surfactant proteins A, B, and D [12, 13] can be observed in PAP patients. Early studies showed that the serum level of LDH is often slightly elevated and maybe a marker of the severity of PAP, but the elevation of tumor markers in PAP was of unclear value [4]. Although tumor markers were widely used in detecting malignancies, they were also detected in nonmalignant diseases. In our data, most patients had an elevation of CEA and all the patients had a raise of cyfra21-1 and NSE. In recent years there were researches studying tumor markers such as CEA, cyfra21-1, and NSE that might reflect the severity of PAP [11, 14, 15]. Compared with other nonspecific lab tests, tumor markers especially cyfra21-1 and NSE may be useful clue in diagnosis and differential diagnosis of PAP.

The pulmonary ventilation function of PAP patients can be normal, but typical results show a restrictive ventilator defect, with a decrease of total lung capacity and vital capacity. PAP patients usually have reduction of diffusing capacity [3, 16]. In present study we found that all the cases showed a decrease of DLCO and after WLL DLCO increased in different degrees. In other words, the diffusing capacity had been improved after WLL. DLCO can be an evaluation indicator of the treatment efficacy.

Radiological examinations are among the most important clues in clinically suspecting and diagnosing PAP for its specific appearance [1, 4, 17]. The plain chest radiograph often reveals bilateral, symmetric, perihilar airspace consolidations in a batwing-like pattern, similar to cardiogenic pulmonary edema but without other signs of heart failure [18]. HRCT shows more specific characteristics than routine chest radiograph. The typical presentation includes patchy, ground-glass opacifications, and consolidation with some thickening of the interlobular and intralobular septa, resulting in the so-called “crazy paving” pattern [4, 19]. Although the crazy paving appearance may sometimes be seen in some other interstitial and airspace lung diseases [20], it is still of important prompt significance. All the patients who were suspected of PAP should receive the HRCT examination in addition to chest radiograph or common CT in order to raise the accuracy of diagnosis.

Open-lung biopsy was considered as “gold criteria” for the diagnosis. However, it is not always required not only because sometimes false negatives are possible for sampling error [4], but also for the reason that diagnosis can be made in about 75 percent of clinically suspected cases by finding the opaque and milky appearing bronchoalveolar lavage fluid which contains large “foamy” macrophages and amorphous, eosinophilic, PAS positive (pink) materials [17, 21]. In our observation, 9 out of 9 cases were confirmed by BALF, 4 out of 5 were confirmed by surgical biopsy, and 1 out of 4 was confirmed by transbronchial biopsy. The results indicated that the diagnosis method through BALF seemed more accurate than transbronchial biopsy. And compared to surgical biopsy, it is more convenient and safe.

Last but not least, we treated 5 cases of PAP patients in local hospital with successively bilaterally lung lavage (one patient was treated with WLL in another hospital and data after WLL were not available). The average lavage volume ranged from 9 L to 15 L for a single lung. Two cases as number 5 and number 8 patients received incomplete single lung lavage, respectively, 1500 mL and 6000 mL in volume because drainage was not good. The 7 cases all improved in clinical symptom, pulmonary diffusing capacity, or CT presentation. According to Table 3, we found that most patients with large capacity alveolar lavage had greater improvement by more than 20% in DLCO when comparing to those with incomplete single lung lavage. It seemed that large volume lung lavage is more effective than halfway lung lavage in treating PAP. But during our follow-up, we found that the two patients who received the lung lavage with less volume relieved much from shortness of breath. On the other hand, we followed up number 3 and number 6 patients, who did not receive WLL, for two years after discharge and found that both were spontaneously relieved according to CT scan and did not suffer from dyspnea any more. So for those who do not have dyspnea or only have slight shortness of breath, WLL may not be necessary. And for those who cannot suffer from large volume lung lavage, less volume lavage also can work and the curative effect makes little difference in the long term.

## Figures and Tables

**Table 1:** Clinical characteristics of PAP patients.

	*n* (%) & median 11
Age, year	51 (30–66)
Gender	
Male	7 (64)
Female	4 (36)
Smoking status	
Never smoker	4 (36)
Current or ex-smoker	7 (64)
Dust exposure	2 (18)
Disease duration, month	8 (0.25–60)
Clinical symptoms	
Asymptomatic	1 (9)
Symptomatic	
Dyspnea	10 (91)
Cough	8 (73)
Chest	2 (18)
Fever	4 (36)
Physical examination	
Inspiratory crackle	6 (55)
Digital clubbing	5 (45)
Cyanosis	2 (18)
Normal	2 (18)

**Table 2:** Clinical examinations of PAP patients.

Patient number	1	2	3	4	5	6	7	8	9	10	11
Laboratory tests											
CRP (mg/L)	3.5	23.3	9.5	1.0	4.7	1.0	21.1	2.7	1.6	6.5	5.0
ESR (mm/h)	22	3	12	16	38	16	37	2.0	2.0	34.0	25.0
PaO_2_ (mmHg)	62.5	48.5	75.5	54.3	59.8	61.2	63.0	81	68.8	62.2	77.4
P(A-a)O_2_ (mmHg)	50.6	140	21.5	50.8	48.2	36.8	42.3	7.9	34.6	31.7	41.0
LDH (U/L)	206	463	244	310	348	279	268	299	233	301	230
CEA (ng/mL)	4.0	13.3	2.3	5.1	8.2	41.8	5.6	45.0	6.0	6.9	4.0
Cyfra 21-1 (ng/mL)	7.1	33.8	6.9	21.6	12.8	12.7	13.0	NA	NA	NA	NA
NSE (ng/mL)	17.5	24.4	17.6	15.0	19.0	21.5	24.0	21.0	NA	NA	NA
Lung function											
FEV1/FVC (%)	94.3	89.9	94.3	91.1	74.2	80.9	88.6	85.7	NA	95.4	100
TLC (% predicted)	65.3	60.4	71.8	46.4	54.2	64.2	78.5	89.6	NA	60.1	65.1
FRC (% predicted)	62.7	60.0	92.2	51.7	42.8	42.8	74.2	112	NA	37.4	46.4
VC (% predicted)	68.0	63.2	73.9	64.8	58.6	89.1	74.4	85.9	NA	62.5	88.9
DLCO (% predicted)	44.0	24.2	40.9	22.4	28.7	56.1	46.6	50.4	NA	36.5	41.7
mMRC score	2	3	1	3	2	0	1	1	3	3	2
Diagnosis method											
Surgical biopsy	+	+	−	+	+	NA	NA	NA	NA	NA	NA
Transbronchial biopsy	NA	NA	NA	NA	NA	−	+	NA	−	−	NA
BALF	+	+	+	NA	NA	+	+	+	+	+	+
Clinical course	R	R	SR	R	R	SR	R	R	S	R	R

NA: not available; R: remission; SR: spontaneous remission; S: stabilization; mMRC: modified British medical research council score for shortness of breath.

**Table 3:** Therapeutic evaluation of WLL.

Patient number	1	2	4	5	7	8	10	11
Before WLL								
DLCO (%)	44.0	24.2	22.4	28.7	46.5	50.4	36.5	41.7
PaO_2_ (mmHg)	62.5	48.5	54.3	59.8	63.0	81	62.2	77.4
mMRC score	2	3	3	2	1	1	3	2
WLL (left)	9.74 L	NA	10.9 L	—	13 L	—	12 L	13 L
WLL (right)	9.8 L	NA	9 L	1.5 L	13 L	6 L	15L	12 L
After WLL								
DLCO (%)	45.0	NA	36.8	30.1	56.0	51.6	48.6	59.3
PaO_2_ (mmHg)	63.2	NA	61.2	77.4	95.6	83.5	78.3	80.3
mMRC score	1	2	2	1	1	1	1	1

NA: not available; mMRC: modified British medical research council score for shortness of breath.
